# Reply to Kempf et al

**DOI:** 10.1093/infdis/jiw498

**Published:** 2017-01-25

**Authors:** L. Thorne, I. Goodfellow

**Affiliations:** 1 Division of Virology, Department of Pathology, University of Cambridge, Addenbrookes Hospital, United Kingdom

To the Editor—In their correspondence, Kempf et al report the use of nitazoxanide, a broad-spectrum antimicrobial agent, for the treatment of a chronic norovirus infection in an immunocompromised patient with X-linked agammaglobulinemia. Such individual case reports are valuable because of the lack of approved antivirals for norovirus infections and the increasing reports of enhanced norovirus-associated morbidity and mortality in immunocompromised patients [1].

Nitazoxanide has previously been shown to reduce the duration of symptoms in immunocompetent individuals and to clear infection in a single immunocompromised patient with chronic myeloid leukemia [2]. In contrast, despite noting an initial improvement in diarrhea frequency and severity, Kempf et al report that nitazoxanide treatment failed to eradicate norovirus infection in their patient. Several follow-up experimental treatments also failed to clear the infection, including ribavirin, which has been reported to have mixed success in the treatment of chronic norovirus infections [3].

The mechanism of action of nitazoxanide against norovirus is currently unknown. For other viruses, it has been shown to be 2-fold, involving inhibition of cellular processes that are required for viral infection and potentiation of the innate immune response [4]. Therefore, the disparity among the results of these individual reports suggests that the success of nitazoxanide treatment could be both virus and host specific and, possibly, influenced by the immune status of the patient. To compare these factors, it is essential that future studies of this kind report the genotype and, ideally, the entire viral genome sequence of the infecting virus, alongside a comprehensive description of the patient's immune status. This additional information will prove invaluable in determining which factors may influence the likelihood of success of the experimental treatments.

Until recently, the lack of a robust and reproducible cell culture system for human noroviruses has presented a major barrier to the development and characterization of antivirals and molecular studies of human norovirus replication. Two recent advances now offer the opportunity to further investigate potential therapeutic approaches and potential strain-specific resistance phenotypes. The demonstration that immortalized B cells allow for limited norovirus replication in cell culture [5] provides one such experimental system. However, the breakthrough development of a stem-cell–derived human enteroid system that supports the replication of multiple human norovirus strains [6] now provides the opportunity to determine the molecular mechanism of action of nitazoxanide and whether its activity is genotype specific. This system also holds great potential for research and development of other, more-effective antiviral treatments for acute and chronic human norovirus infections.
